# Armed conflict, alcohol misuse, decision-making, and intimate partner violence among women in Northeastern Uganda: a population level study

**DOI:** 10.1186/s13031-018-0173-x

**Published:** 2018-08-06

**Authors:** Jennifer J. Mootz, Florence Kyoheirwe Muhanguzi, Pavel Panko, Patrick Onyango Mangen, Milton L. Wainberg, Ilana Pinsky, Kaveh Khoshnood

**Affiliations:** 1 Department of Psychiatry, Columbia University, 1051 Riverside Drive, New York, NY 10032 USA; 2 New York State Psychiatric Institute, 1051 Riverside Drive, Kolb 171, New York, NY 10032 USA; 3 Department of Women and Gender Studies, Makerere University, P.O. Box 7062, Kampala, Uganda; 4 Department of Educational Psychology and Leadership, Texas Tech University, 3008 18th Street, Lubbock, TX 79409 USA; 5 Transcultural Psychosocial Organization Uganda, Plot 3271 Kansanga off Ggaba Road, P.O.Box 21646, Kampala, Uganda; 6 School of Public Health, Yale University, 60 College St, New Haven, CT 06510 USA

**Keywords:** Armed conflict, Uganda, Alcohol use, Domestic violence, Decision-making

## Abstract

**Background:**

Relations among and interactions between exposure to armed conflict, alcohol misuse, low socioeconomic status, gender (in)equitable decision-making, and intimate partner violence (IPV) represent serious global health concerns. Our objective was to determine extent of exposure to these variables and test pathways between these indicators of interest.

**Methods:**

We surveyed 605 women aged 13 to 49 who were randomly selected via multistage sampling across three districts in Northeastern Uganda in 2016. We used Mplus 7.4 to estimate a moderated structural equation model of indirect pathways between armed conflict and intimate partner violence for currently partnered women (*n* = 558) to evaluate the strength of the relationships between the latent factors and determine the goodness-of-fit of the proposed model with the population data.

**Results:**

Most respondents (88.8%) experienced conflict-related violence. The lifetime/ past 12 month prevalence of experiencing intimate partner violence was 65.3%/ 50.9% (psychological) and 59.9%/ 43.8% (physical). One-third (30.7%) of women’s partners reportedly consumed alcohol daily. The relative fit of the structural model was superior (CFI = 0.989; TLI = 0.989). The absolute fit (RMSEA = 0.029) closely matched the population data. The partner and joint decision-making groups significantly differed on the indirect effect through partner alcohol use (*a*_*1*_*b*_*1*_ = 0.209 [0.017: 0.467]).

**Conclusions:**

This study demonstrates that male partner alcohol misuse is associated with exposure to armed conflict and intimate partner violence—a relationship moderated by healthcare decision-making. These findings encourage the extension of integrated alcohol misuse and intimate partner violence policy and emergency humanitarian programming to include exposure to armed conflict and gendered decision-making practices.

## Background

In 2013, the World Health Organization, United Nations Development Programme, and partners joined to address the intersections of alcohol misuse, gender-based violence, and infectious diseases, seminally acknowledging the interrelatedness of these problems at a policy level [1]. This policy momentum has not explicitly addressed exposure to armed conflict, however, and there remains a paucity of research that examines these problems beyond bivariate relations and as conceptually interrelated, signifying a policy-to-research gap. *Wicked problems* involve numerous stakeholders and participants, result from intersecting trends, are embedded in other wicked problems, and cannot be easily solved [2]. Relations among and interactions between exposure to armed conflict, alcohol misuse, low socioeconomic status, gender (in)equitable decision-making, and intimate partner violence (IPV) represent such problems. All are of serious global public health concern not only because of their scope and impact presently but also because of their enduring and intergenerational effects.

In 2015, armed conflicts forcibly displaced 65.3 million refugees or asylum seekers worldwide [3], with the bulk of these displacements affecting civilians in low-income countries [4]. The negative health and mental health outcomes of exposure to armed conflict are numerous and long lasting [5, 6]. For instance, conflict-affected adolescents in Uganda have reported experiencing symptom constellations consistent with depression and anxiety [7]. Cumulative exposure to potentially traumatic events have corresponded to higher rates of mental illness [5], consistent with a dose-response effect. However, the pathway between exposure to traumatic events and poor mental health may be indirect and partially mediated by increased exposure to adverse daily stressors, such as poverty and marginalization [8]. For example, In addition to threats to loved ones and deaths, material losses significantly predicted poor functioning in former child solders in Uganda [9] . Illustrating temporal impact, in Liberia the distribution of posttraumatic stress symptoms has mapped geographically onto areas affected by the conflict almost two decades after civil war [10]. Poor mental health can in turn relate to perpetration of and endorsement of violence [11]. In Uganda, for instance, conflict-affected civilians who met symptom criteria for PTSD were more likely to identify violent resolutions as means to achieve peace [12]. Finally, armed conflict exposure has protractedly negatively affected development and progress on the United Nation’s Millennium Development Goals by increasing rates of malnutrition and infant deaths and reducing access to clean drinking water [13].

As another form of interpersonal violence, intimate partner violence (IPV), defined as intimate partner behaviors that cause physical, psychological, or sexual harm [14] is likewise wide-reaching, with nearly three out of four women reporting experiencing sexual or physical abuse by an intimate partner in some Sub-Saharan African countries [15]. Acts of IPV escalate in armed conflict settings and are often times more frequent than other forms of gender-based violence in these settings [16]. Like exposure to armed conflict, IPV ensues health, mental health, and intergenerational sequelae [17]. There is a strong relation between mothers’ mental health and their children’s mental and physical wellbeing, for instance [18]. Witnessing IPV as a child is a risk factor for men’s perpetration of IPV later in life [19].

Representing another wicked problem, alcohol misuse is the fifth leading cause of disability and premature death globally and is the leading cause of the aforementioned for people ages 15 to 49 [20] The association between alcohol misuse with the perpetration of IPV is well established, including in sub-Saharan Africa [21, 22]. IPV severity has been associated with level of alcohol consumption [23]. The type of misuse seems to matter with heavy episodic drinking presenting an association with perpetration and victimization of IPV. Recent studies have established a temporal relation with acute intoxication often preceding IPV [24]. In a qualitative study conducted with formerly abducted women in the Lord’s Resistance Army, every reported case of IPV included discussion of male alcohol use [25]. Another study from the same region of Uganda found that in addition to women’s exposure to war and re-experiencing symptoms, men’s level of alcohol use significantly predicted IPV [26].

What remains understudied is whether experiences of armed conflict associate with alcohol misuse and mechanisms or risk factors through which this relation might occur [27]. Increased alcohol use has corresponded to trauma exposure and negative mental health symptoms, especially for men [28, 29]. A review of 22 studies with conflict-affected civilians found male gender, cumulative trauma exposure, depression, and older age as correlates with harmful alcohol use [27]. In an internally-displaced population in the Republic of Georgia, exposure to traumatic events and depressive symptoms associated with alcohol use with 28% of men endorsing problematic drinking patterns in contrast to 1% of women endorsing the same [30]. In comparison to Ugandan displaced women, male displaced persons were almost sevenfold likely to have developed alcohol disorder following exposure to conflict [31]. In addition to traumatic experiences of witnessing or fearing loss of life during conflict, economic adversity during and post conflict could escalate alcohol consumption. For instance, because unemployment is high in conflict-affected communities, men may feel that they have failed to meet their gender role expectation of provider and drink alcohol to pass the time [32]. A finding from 33 countries demonstrated that riskier single occasion drinking associated with lower socioeconomic status in men in low-income countries [33].

### The present study

This study took place in the Teso Subregion of Northeastern Uganda, a rural region that has been affected by protracted armed conflict since the 1940’s wherein the Karamajong have been perpetrating cattle rustling raids (violent raids that loot livestock) that have perpetuated instability in Teso [34, 35]. Teso was additionally subjected to atrocities perpetrated by the rebel Lord’s Resistance Army in 2003 [35]. Beginning in 2006, the Ugandan government has executed a now largely successful disarmament campaign in Karamoja, although sporadic cattle raids still occur. Uganda, one of the poorest countries in the world [36], has some of the uppermost rates of IPV [37]. Per capita alcohol consumption in Uganda is one of the highest in sub-Saharan Africa with over 12% of alcohol users engaging in heavy episodic drinking and almost 90% of the consumption consisting of strong, unregulated home-distilled alcohol [38]. Roughly 10% of Ugandan adults have reported levels of alcohol consumption that constitute an alcohol use related disorder [39].

**Fig. 1:**
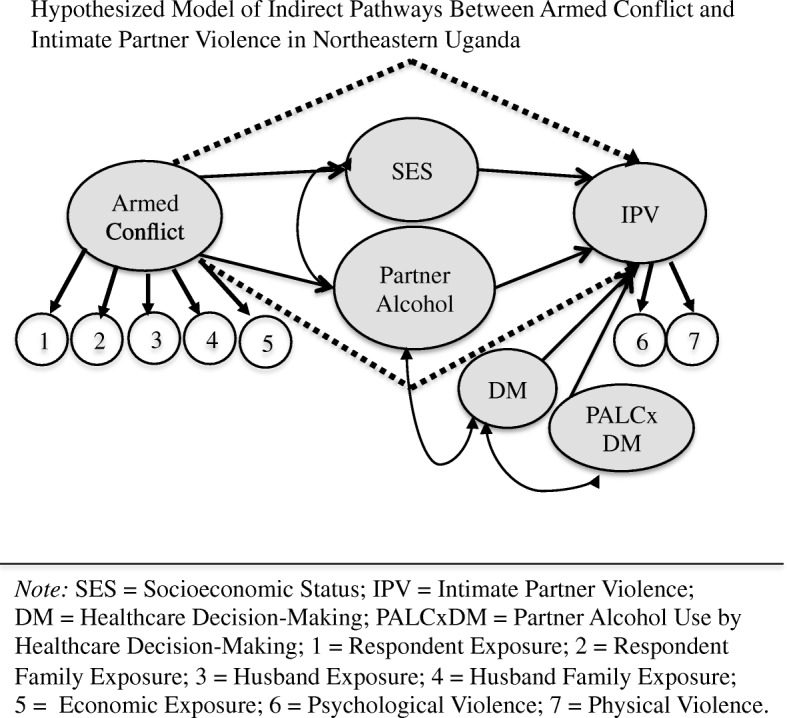
Hypothesized Model of Indirect Pathways Between Armed Conflict and Intimate Partner Violence in Northeastern Uganda

Our objective was to determine extent of exposure to alcohol misuse, low socioeconomic status, gender (in)equitable decision-making, intimate partner violence, and armed conflict and test pathways between these indicators of interest. We hypothesized that (1) exposure to armed conflict would lower socioeconomic status (SES) and increase partner alcohol consumption, both of which would (2) negatively correlate and (3) constitute indirect pathways between exposure to armed conflict and psychological and physical and IPV (Fig. 1).

Not all male partners who consume alcohol to intoxication perpetrate IPV, and the problem of how best to explain the variance remains. We utilized local explanations from a prior qualitative study suggesting that male partner alcohol use intersected with relational exchanges over unequally distributed resources prior to IPV [40]. The Gender Inequality Index—assessing inequality in the labor market, empowerment, and reproductive health—shows Uganda as ranking on the lower end globally in terms of equality [41]. In 2011, 23% of Ugandan women reported making decisions about their own healthcare, only a 1% increase from 2006 [41]. Thus, we additionally hypothesized that (4) gender (in)equitable decision-making would moderate the relationship between partner alcohol misuse and exposure to IPV— differences would exist between women who made their own healthcare decisions, whose partners made women’s healthcare decisions, and who made healthcare decisions jointly with their partners.

## Method

The proposed project was a collaborative one between two academic institutions, one based in the U.S. and one in Uganda, as well as a partnership with a local nonprofit organization called Transcultural Psychosocial Organization (TPO) Uganda. The collaborators jointly constructed the project’s scientific aims, rationale, and the study design.

### Instrumentation

Survey questions were forward translated into Ateso and back translated into English by two separate people who are native speakers of Ateso, from the Teso Subregion, and have postgraduate training in English literature and translation. The committee of translators and first author discussed any discrepancies in translation until consensus was reached.

#### Demographics, partner alcohol use, and intimate partner violence

Demographics, alcohol use, and intimate partner violence were assessed with a modified *Survey of Women’s Health and Life Experiences in Uganda: Woman’s Survey* [42]. Demographics included age, partner status, education level, number of children, and resource ownership, among others. Socioeconomic status (SES) was determined by number of household resources, literacy, and whether or not the partner was working.

Non-clinically diagnostic partner alcohol use was evaluated with three questions assessing three domains: frequency of drinking, frequency of appearing inebriated in the past 12 months or the last 12 months of the relationship, and problematic impact of alcohol use—assessment questions congruent with the World Health Survey conducted in 20 African countries with over 75,000 adults [43], international guidelines [44], and brief alcohol use questionnaires [45]. Additional items assessed whether partners reportedly consumed alcohol when he was unable to find work and why participants perceived IPV events to occur. A final item asked whether respondents thought partners were stressed/depressed/angry (non-clinical constructs intended to assess perceptions of partners’ generalized distress) because of lack of work opportunities or low income.

The outcome variables of intimate partner psychological and physical violence were measured with standard World Health Organization items of violence against women and girls. Items were demonstrative of specific aggressive behaviors, such as harassing, slapping, hitting, and cutting (*No* or *Yes*), that occurred across the lifetime and within the past 12 months [46]. Adaptations of this instrument included omitting unrelated items. The IPV items demonstrated an internal consistency of *α* = 0.88.

#### Exposure to armed conflict

The team adapted the *Exposure to Political Violence Inventory* [45] to address the unique sociopolitical context of Teso (24 items). Exposure experiences included verbal, physical, sexual, relocation, abduction, loss of life, property theft and damage, and work lost for participants, partners, and family members of both. Respondents denoted whether they had experienced the event (*No* or *Yes*). This measure was preferable because its items closely assessed problems associated with cattle raiding, including economic loss, which emerged as a key variable in pathways between armed conflict and gender-based violence in previous qualitative research [47]. Notable adaptations consisted of adding sexual assault, abduction of self or children, and having livestock stolen, an importance source of sustenance in Teso. Internal consistency of the adapted measure was *α* = 0.88.

#### Decision-making practices

We used an item from the *International Men and Gender Equality Survey: Women’s Questionnaire* [48] to assess decision-making practices in the household. The following indicators of who made women’s healthcare decisions were as follows: *Yourself, Partner, Jointly, Someone Else, Yourself and Someone Else*. For analysis, we collapsed the five options into three groups by combining *Someone Else* with *Partner* to indicate decisions were made by partner or someone else and *Yourself and Someone Else* with *Jointly* to indicate joint decision-making with partner or someone else.

### Procedure

TPO Uganda identified six bilingual research team members. Following institutional review board approval in the U.S. and locally, the first author facilitated a three-day training for the research team, reviewing ethical principles of data collection—including the process of informed consent for adults and emancipated adolescents, considerations for surveying intimate partner violence study procedures, and safety assessment and protocol processes [49, 50], which were already in place for another large research study and managed by TPO Uganda. The research team piloted the survey administration, after which they reconvened to review instruments item by item for local understandability and consensually make needed modifications.

We employed a multistage sampling strategy to ensure a range of experiences with conflict and make the results generalizable to the population in the surveyed districts. Our local partners purposively selected three districts in Teso based on the anecdotal frequency and severity of exposure to the Karamajong raids and cattle theft: Katakwi District as representative of high exposure, Amuria District as medium, and Kumi District as low. The team identified subcounties within each district that represented the district’s exposure level; eight villages were randomly selected, and a minimum of 25 participants was surveyed in each village (*n* = 605). Almost half of the population of Teso (roughly 2.5 million) was ineligible due to age [51] and another half ineligible because of gender. We calculated the sample size from the remaining with a 95% Confidence Level, indicating a required sample size of 385 (https://www.calculator.net/sample-size-calculator.html). Additional sample data were obtained to increase statistical power and allow for structural equation modeling analyses. The research assistants formed three teams of two people each, started in the geographic center of each village, and spun a pen to sample every third household. Eligible participants included all women aged 18 to 49 and married (self-defined by participants and including legal or customary) girls under age 18. The number of approached households was 696. Of these, 20 women declined to participate, four households spoke a language other than Ateso, and 67 households had no eligible women or inhabitants were absent, leaving a response rate of approximately 87%.

Research team members solicited the given names of eligible participants who were present and randomly selected one participant. If the selected participant agreed to be interviewed, then the research pair asked the participant to identify a place for privacy. Once privacy was attained, the research pair read the verbal (due to a high illiteracy rate) consent script. To conclude, the research pair gave all participants a list of resources and a bar of soap— determined by our local partners.

### Data analysis

We used SPSS 24 to establish frequencies. We used Mplus 7.4 to estimate a structural equation model for currently partnered women (*n* = 558) to evaluate the strength of the relationships between the latent factors and determine the goodness-of-fit of the proposed model with the population data. We assessed fit using the comparative fit index (CFI; [52]), the Tucker-Lewis index (TLI; [53]), and the root mean square error of approximation (RMSEA; [54]). The CFI and TLI measure relative model fit by comparing the proposed model to the worst possible fitting model. The greater the improvement demonstrated by the proposed model, the closer CFI and TLI approach 1— superior fit is typically set at .95. The RMSEA is a measure of absolute fit and quantifies model misspecification; it indicates better fit as the value approaches 0. The typical threshold for close fit is .05 [55]. Due to the categorical nature of the manifest indicators in the model, we used the weighted least squares means and variances method to estimate the correlations for the ordinal and binary items [56].

Due to the presence of multiple intermediary predictors, we used a parallel indirect effects model. The hypothesized indirect effects, operationalized as the product of each intermediary variable’s *a* and *b* paths, were examined as a standard mediation. We used bias-corrected bootstrapping methods with 5000 iterations to test the significance of the indirect effect parameters with a 95% confidence interval [57]. The indirect effect is significant if the confidence interval does not cross 0.

Finally, we theorized the indirect effects to differ across groups of household decision-making (respondent [r], jointly [j] or partner [p]). The multiple group framework was utilized to estimate whether there was a significant difference in the indirect effects based on decision-making group. The procedure involved testing measurement invariance, estimating the indirect effects separately for each group, and determining whether their differences were significant using bootstrapped confidence intervals.

## Results

### Respondent and partner characteristics

Participants’ ages ranged from 13 to 49 (*M* = 29.88; see Table 1). Most women were currently partnered (92.8%). The majority of girls and women were illiterate (60.3%), many indicated having no schooling (14.7%), and most endorsed having only some primary education (averaging approximately 4.5 years of education), which contrasted with their male partners who were more likely to be able to read and write (82.2%), have had some schooling (94.3%), and had a higher level of schooling (roughly averaging 7.5 years of education). Respondents had four children on average. The majority of women identified as Catholic (46.0%) or Anglican (45.4%).

**Table 1:** Respondent and partner characteristics in Northeastern Uganda

Variable	Katakwi District (*n* = 202)	Amuria District (*n* = 201)	Kumi District (*n* = 202)	All (*n* = 605)
Age (years)				
Respondent				
*M (SD)*	30.28 (9.09)	29.33 (9.05)	30.01 (8.55)	29.88 (8.89)
Range	17–49	13–49	18–49	13–49
Partner				
*M (SD)*	36.2 (11.28)	34.57 (10.77)	36.16 (11.30)	35.68 (11.13)
Range	18–74	18–65	19–78	18–78
Partner Status (%)				
Currently partnered	93.9	94.1	90.6	92.8
Ever partnered	4.0	5.0	8.4	5.8
Number Children				
*M (SD)*	4.08 (2.29)	4.49 (2.68)	3.90 (2.45)	4.15 (2.48)
Range	1–11	0–11	0–13	0–13
Education				
Respondent				
No education (%)	18.9	13.9	11.4	14.7
Education years *M (SD)*	4.05 (3.09)	4.43 (2.71)	5.06 (3.49)	4.51 (3.14)
Partner				
No education	4.7	3.6	9.0	5.7
Years education	7.87 (3.13)	7.06 (2.57)	7.40 (3.30)	7.43 (3.01)
Literacy (% literate)				
Respondent	34.3	44.3	40.1	39.7
Partner	87.2	84.8	74.9	82.2
Religion				
No religion	0.5	0.0	0.0	0.2
Born again	4.4	6.0	8.4	6.3
Islam	0.0	0.5	0.5	0.3
Catholic	48.8	61.2	25.7	45.4
Anglican	43.8	30.3	63.4	46.0
Other	2.0	1.5	2.0	1.8

### Prevalence rates of exposure to IPV and armed conflict and partner alcohol use

The respective lifetime/ past 12 months prevalence of respondents who reported experiencing psychological violence was 65.3%/ 50.9% and physical violence was 59.9%/ 43.8% with 71.9%/ 56.2% of women being exposed to either form of violence (see Table 2). Exposure to armed conflict was 72.1% for respondents, 55.9% for respondents’ families, 72.4% for partners, and 35.9% for partners’ families. Experience of economic loss due to conflict was 64.2%. Most respondents (88.8%) divulged experiencing some type of conflict-related violence or loss for themselves or partners.

**Table 2:** Exposure rates to intimate partner violence and armed conflict in Northeastern Uganda

Variable	Katakwi District (*n* = 202)	Amuria District (*n* = 201)	Kumi District (*n* = 202)	All (*n* = 605)
Intimate Partner Violence (%)				
Lifetime				
Psychological	69.4	66.0	60.4	65.3
Physical	63.4	61.4	55.1	59.9
Any	74.5	70.9	70.4	71.9
Past 12 Months				
Psychological	57.9	52.2	42.6	50.9
Physical	49.5	46.3	35.6	43.8
Any	61.4	56.2	51.0	56.2
Armed Conflict (%)				
Respondent	83.7	90.8	41.3	72.1
Husband	80.2	85.8	46.4	72.4
Respondent family	64.8	59.7	42.7	55.9
Husband family	43.4	35.7	27.9	35.9
Economic	90.9	62.3	38.0	64.2
Any	96.0	94.8	73.8	88.8

About one-third (30.7%) of women’s partners reportedly consumed alcohol daily or nearly daily with a comparable frequency of drinking to intoxication on most days (34.3%). Fewer partners consumed alcohol once or twice per week (21.3%), and many partners rarely or never consumed alcohol (46%) with a slightly higher percentage (52.5%) rarely or never drinking to intoxication. Almost half of the respondents (47.7%) positively endorsed at least one of the items, indicating their partners’ level of alcohol consumption as negatively affecting family relations (29.9%), financial resources (6.8%), or other problems (11%). Most women (56.4%) indicated that their partners drank because of unemployment, and a very close percentage (56%) thought their partners were stressed, depressed, or angry because of lack of employment opportunities or low income. Of women who had experienced IPV, 35.1% believed that the abuse occurred because their partner was inebriated.

### A moderated model of indirect pathways between armed conflict and IPV

**Fig. 2:**
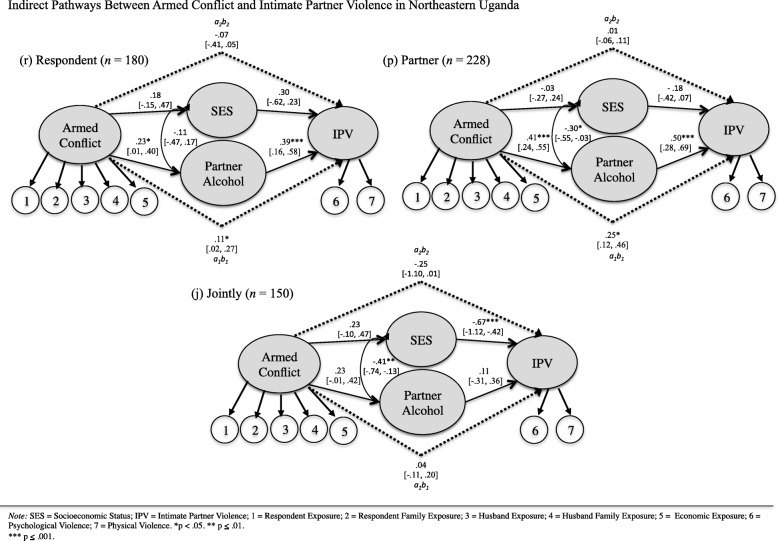
Indirect Pathways Between Armed Conflict and Intimate Partner Violence in Northeastern Uganda

The hypothesized model passed both the weak and strong levels of measurement invariance (see Table 3). The relative fit was superior (CFI = 0.989, TLI = 0.989), and the absolute fit closely matched (RMSEA = 0.029) the population data. Per Fig. 2, the indirect effect of armed conflict on IPV through the influence of partner alcohol use was significant for the respondents (*n* = 180) who made their own healthcare decisions (group r; *a*_*1*_*b*_*1*_ = 0.108 [0.021: 0.271]), as well as for women (*n* = 228) whose partner made their healthcare decisions (group p; *a*_*1*_*b*_*1*_ = 0.252 [0.120: 0.460]), but not for women (*n* = 150) who made household decisions jointly with their partners (group j; *a*_*1*_*b*_*1*_ = 0.043 [− 0.108: 0.195]). On the other hand, the effect of armed conflict on IPV passing through SES was not significant for the respondent group (*a*_*2*_*b*_*2*_ = − 0.066 [− 0.410: 0.047]) or the partner group (*a*_*2*_*b*_*2*_ = 0.006 [− 0.060: 0.110]), although it approached significance for the joint group (*a*_*2*_*b*_*2*_ = − 0.252 [− 1.10: 0.014]).

**Table 3:** Measurement invariance results

Model	χ^2^(df)	RMSEA(CI)	CFI	TLI	ΔCFI	Held?
Configural	1499.60(1234)	0.030(0.021:0.036)	0.989	0.988	–	Yes
Weak	1504.85(1298)	0.029(0.022:0.036)	0.99	0.988	−0.001	Yes
Strong	1609.71(1362)	0.031(0.024:0.037)	0.988	0.987	0.002	Yes

*Note. RMSEA* root mean square error of approximation, *CI* confidence interval, *CFI* comparative fit index, *TLI* Tucker-Lewis index

Although the respondent (r) and partner (p) groups as well as the respondent (r) and joint (j) groups did not differ on either of the indirect effects, the partner (p) and joint (j) groups significantly differed on the indirect effect through partner alcohol use (*a*_*1*_*b*_*1*_ = 0.209 [0.017: 0.467]). Thus, in the partner group (p), armed conflict affected partner alcohol use, which in turn predicted IPV to a significantly higher degree than in the joint group (j). The partner (p) and joint (j) groups additionally approached significance regarding the indirect effect passing through SES (*a*_*2*_*b*_*2*_ = 0.258 [− 0.015: 1.105]).

## Discussion

The purpose of this study was to structurally test pathways and interactions among five wicked public health problems: exposure to armed conflict, alcohol misuse, low socioeconomic status, gender (in)equitable decision-making, and psychological and physical IPV. A moderated structural regression model illustrating indirect pathways inclusive of partner alcohol misuse and socioeconomic status from exposure to armed conflict to IPV fit the population data superbly. To our knowledge, this is the first study to quantitatively examine these five problems as conceptually related in a cohesive quantitative model.

For women who made healthcare decisions independently and women whose partners made the healthcare decisions for women, the indirect pathways connecting partner alcohol misuse to exposure to armed conflict and IPV were significant. In contrast, the partner alcohol misuse pathway was not significant for respondents who made their healthcare decisions jointly with partners. There were significant differences between healthcare decision-making groups: Women whose partners held more decision-making power in the household and consumed alcohol were more likely to experience IPV than women who made healthcare decisions jointly with partners. Significant differences between the group of women who made their own healthcare decisions and women whose partners made those decisions did not manifest. Thus, it could be that the psychopharmacologic effects of alcohol interact with relational negotiation of resources where decision-making is inequitable in either direction. Some men may experience heightened motivation to maintain previously existing dominant structures or to re-establish these structures (i.e., the violence backlash) when they perceive it as being threatened.

In contrast to the respondent and partner decision-making groups, IPV was significantly associated with SES in the joint decision-making group. Recent research has tested interactions between acceptance of IPV and socioeconomic variables, leading to the development of a “gendered resource theory” [58], which suggests that the acceptability of IPV moderates the effect of SES variables on the occurrence of IPV. Our results show that in relationships where decisions about healthcare resources are shared, and male partner alcohol use is controlled for, SES plays a larger role in IPV. Our findings demonstrate the relation between SES and IPV is increasingly nuanced.

### Implications for policy, programming, and future research

Our findings highlight several potential avenues for policy programming globally. Rates of exposure to armed conflict and IPV were high—a poignant reminder of the scale of the problem. Alcohol misuse rates were also elevated compared to other findings from Uganda where 12.7% were identified as high-end alcohol users [39], although comparable or even less than other studies with male conflict-affected populations [27]. Effectiveness of structural level interventions for the reduction of alcohol misuse and concomitantly, IPV, in low- and middle-income countries is scant. Several top-down interventions targeting alcohol consumption are underway in Uganda with some districts instituting bylaws restricting opening hours for bars. There is stronger evidence to support the effectiveness of structural interventions in low- and middle-income countries aimed at reducing IPV [59, 60]. Our results suggest that policy makers should extend collaborative policy efforts for alcohol misuse and IPV to not only conceptualize these problems as interrelated, but also include exposure to conflict and resource decision-making in households as further interacting problems.

Clinically, an important step forward will be to integrate alcohol misuse and IPV treatment—a rarely endeavored strategy in settings at all levels of development, but particularly in LMIC. While WHO and UNHCR have developed guidelines for the treatment of substance use disorders in humanitarian settings [61], a systematic review found that no interventions for substance use have been tested with conflict-affected populations [27]. Another review of 22 studies (only one of which took place in an LMIC) examining the impact of alcohol interventions at all socioecological levels on IPV found the evidence base to be “disappointingly small” ([62]; p. 888). However, promising evidence suggests that integrated group dual treatments targeting both alcohol misuse and IPV are more efficacious in reducing both alcohol use and IPV over treatments addressing alcohol misuse only [63].

Although men are at higher risk for developing alcohol misuse problems in relation to exposure to conflict and trauma, and in low-income settings have higher risk of IPV perpetration, integrated approaches to comorbid problems often utilize women as research participants or treatment subjects. In a review of 71 research studies targeting integrated HIV/IPV as risk factors, all studies were conducted with women, and the authors concluded that more interventions focusing on men and male risk were needed [64]. Negative mental health experiences could be related to a stronger likelihood to use alcohol to manage negative affect related to conflict. In post-conflict Rwanda, IPV perpetrators were more likely to endorse having mental health problems than were victims, for instance. Emergency humanitarian actors should target men for integrated substance use prevention and response programming and consider the role of substance use in IPV. However, more research with conflict-affected men is needed to better understand the variables involved in indirect pathways between armed conflict and IPV for intervention.

### Limitations

Some limitations should be considered when interpreting the results. The multistage design can lead to higher sampling error, for instance. Next, women secondarily reported their male partners’ levels of alcohol consumption and exposure to armed conflict, a strategy shown to produce reliable data in other contexts [65], but could have resulted in bias. Tensions such as this are inherent in research with IPV: Men might underreport levels of perpetration, and it is ethically counter-indicated to gather data from couples because of concerns about inadvertently increasing IPV. Furthermore, while the alcohol use questions are congruent with those in other widely used surveys [45], no study of which we are aware has validated these in Uganda. Finally, the data are cross-sectional, limiting our ability to draw conclusions about causality. Though it is challenging to obtain pre-conflict baseline data, more longitudinal research is needed to confirm these findings.

## Conclusion

Exposure to armed conflict, alcohol misuse, low socioeconomic status, gender (in)equitable decision-making, and intimate partner violence behaviors are wicked problems observed with high frequency in this study in Northeastern Uganda. Our research highlights an indirect pathway through male partner alcohol use that connects IPV to armed conflict for women who make their own healthcare decisions and for women whose partners make those decisions, showing important interactions. We recommend the integration of alcohol misuse, IPV, household decision-making practices policy and interventions for men in conflict-affected settings.
